# NHWD-870 protects the kidney from ischemia/reperfusion injury by upregulating the PI3K/AKT signaling pathway (experimental study)

**DOI:** 10.25122/jml-2022-0309

**Published:** 2023-06

**Authors:** Saba Sahib Younis, Fadhaa Abdul Ameer Ghafil, Sahar Majeed, Najah Rayish Hadi

**Affiliations:** 1 Al-Sadr Medical City, Al-Najaf Health Directorate, Al Najaf Al-Ashraf, Iraq; 2 Department of Pharmacology & Therapeutics, Faculty of Medicine, University of Kufa, Kufa, Iraq.

**Keywords:** NHWD-870, ischemia reperfusion injury, PI3K/AKT, IL-1B, BCL-2, PGF-2, AKI: Acute Kidney Injury, BCL-2: B-cell lymphoma-2, BUN: Blood Urea Nitrogen, DMSO: Dimethyl Sulfoxide, ELISA: Enzyme-Linked Immunosorbent Assay, I/R: Ischemia Reperfusion, IL-1B: Interleukin-1 Beta, P-Akt: Phosphorylated-Protein Kinase B, PI3K: Phosphatidylinositide 3-Kinases, RIRI: Renal Ischemia-Reperfusion Injury, ROS: Reactive Oxygen Species, SCr: Serum Creatinine

## Abstract

Renal ischemia-reperfusion injury is a critical clinical condition with a potentially fatal prognosis if not adequately managed. NHWD-870, a known Brd4 inhibitor with anti-cancer properties, exhibits additional attributes such as antioxidant, anti-inflammatory, and anti-apoptotic effects, suggesting its potential to preserve renal tissue and mitigate damage during ischemic insults. We aimed to assess the potential nephroprotective effect of NHWD-870 by investigating its anti-apoptotic, anti-inflammatory, and antioxidant properties in a rat model of renal ischemia-reperfusion injury. Male Wistar Albino rats (n=24) were randomly assigned to four groups: sham, control, vehicle, and NHWD-870. The control group experienced bilateral renal ischemia for 30 minutes, followed by 2 hours of reperfusion, while the sham group underwent a laparotomy without ischemia-reperfusion induction. The vehicle group received a DMSO injection, and the NHWD-870 group was administered 3mg/kg NHWD-870 orally 24 hours before repeating the control group protocol. Blood samples were collected after reperfusion for blood urea nitrogen (BUN) and serum creatinine (SCr) analysis. ELISA method was used to assess IL-1B, BCL-2, PGF-2, and PI3K/AKT signaling pathways in renal tissue. Tubular injury severity was evaluated through histopathological analysis. NHWD-870 treatment improved renal function and histological preservation compared to the control and vehicle groups. BUN, sCR, IL-1B, BCL-2, and PGF-2 levels in renal tissue were significantly improved in the NHWD-870 group (p<0.05). Furthermore, the PI3K/AKT signaling pathway was significantly upregulated (p<0.01), and tubular injury severity was reduced in the NHWD-870 group. NHWD-870 demonstrated substantial nephroprotective effects in reducing renal damage induced by ischemia-reperfusion injury in rats. These effects may be attributed to the anti-apoptotic properties, as indicated by increased levels of the anti-apoptotic protein Bcl-2, and the reduction in oxidative stress marker PGF-2 through upregulation of the PI3K/AKT signaling pathway, along with the decrease in the inflammatory marker IL-1B.

## INTRODUCTION

Ischemia-reperfusion injury (IRI) is a sudden temporary reduction in blood and oxygen levels (hypoxia or anoxia), followed by restoring blood flow and re-oxygenation. This process leads to oxygen and nutrient depletion, resulting in detrimental effects such as inflammation and oxidative stress [1]. Acute kidney injury (AKI) is a condition often caused by reperfusion damage, and it is associated with increased mortality and morbidity in hospitalized patients [2]. Renal ischemia-reperfusion injury can occur in clinical settings such as renal transplant, renal artery angioplasty, aortic bypass, cardiopulmonary bypass, nephrectomy surgery, and iatrogenic injury [3]. The severity of IRI damage is determined by the tissue's metabolic needs, the duration of the ischemic insult, and the technique and amount of reperfusion [4]. Prolonged ischemia can result in kidney cell death, impaired renal function, and/or the advancement of interstitial fibrosis [5].

Renal ischemia triggers a complex cascade of inflammatory responses, exacerbating renal damage.

Interleukin-1 beta (IL-1B) is an important proinflammatory cytokine that regulates inflammation, usually produced in response to pathogens invasion and tissue damage [6]. IL-1B is a critical pro-inflammatory cytokine that regulates the innate immune response of the host proinflammatory responses by attaching to the type 1 interleukin receptor (IL-1R1). Measuring lipid peroxidation is a way to evaluate the extent of oxidative damage. F2-isoprostane is a reliable biomarker of oxidative stress and is commonly used to evaluate lipid peroxidation in animal and human studies [7]. Isoprostanes are a category of prostaglandins primarily synthesized through a non-enzymatic pathway, independent of the direct involvement of cyclooxygenase (COX) enzymes from arachidonic acid. In both animal and human models of oxidative stress, these molecules are reliable indicators of lipid peroxidation [8]. F2 isoprostane, a recognized biomarker for oxidative stress in various diseases, is generated via lipid oxidation of arachidonic acid, a nonenzymatic pathway activated by free radicals [9]. In ischemia, F2-isoprostanes have been utilized as indicators of oxidative damage. During a partial nephrectomy, measurements of renal isoprostane levels may detect the amount of kidney injury and the effects of ischemia in real time. Renal isoprostane levels have been shown to increase with greater vascular occlusion time and during reperfusion phases in many studies [10].

Apoptosis results from hypoxia stress in ischemic damage during reactive oxygen species production in reperfusion injury. There are two major pathways involved in apoptosis: the extrinsic pathway and the mitochondrial pathway. The extrinsic pathway (death receptor pathway) is stimulated by death ligands and receptors involving TNF-α, TNF related weak inducer of the apoptosis signaling pathway, Fas ligand, which is a type II transmembrane protein belonging to the TNF family, TRAIL, which is TNF -related apoptosis-inducing ligand, and TL1A which is a TNF group member [11]. On the other hand, the mitochondrial pathway of apoptosis is triggered when the mitochondria's outer membrane ruptures, enabling proapoptotic agents (cytochrome c) to escape into the cytosol. This pathway is regulated by the Bcl2 protein family. The members of the B-cell lymphoma 2 (BCL-2) protein family include various members who either encourage cell death or allow their survival [12]. Bcl-2 was discovered as a pro-survival factor produced by follicular B-cell 2 lymphoma cells and was the first (and original) member of the family. Many other Bcl-2 family functions that go well beyond their function as "cell death regulators" have been described in recent investigations [13].

Ischemic stroke, nephrotoxic infection, as well as other renal injuries, such as acute glomerulonephritis, all play a part in the involvement of Bcl-2 proteins in acute renal illness. PI3K/Akt signaling system controls a variety of cellular functions, including cell survival, proliferation, metabolism, motility, and cancer growth. PI3K belongs to the lipid kinase family that may phosphorylate the inositol ring 3'-OH group in inositol phospholipids in the plasma membrane. PI3K and its downstream effector Akt are members of a well-studied family of signal transduction enzymes that regulate cellular activation, inflammation, and apoptosis [14]. NHWD-870 is a potent, orally active, and selective inhibitor of the BET family of bromodomains. This compound can reduce cancer cell-macrophage interactions and exhibits significant tumor-suppressive properties. Its effects extend beyond directly inhibiting tumor growth, and it also prevents tumor-associated macrophage growth in a number of other ways [15]. We hypothesized that NHWD-870 might have a potential nephroprotective effect during renal injury induced by ischemia-reperfusion in a rat model due to its known anti-inflammatory, antioxidant, and antiapoptotic properties via its effect on PI3K/AKT signaling pathway.

## MATERIAL AND METHODS

### Study design

Adult male Wistar Albino rats weighing 250-350 gr, aged 16-24 weeks, were included in this study. The rats were kept in an animal facility at the College of Science, University of Kufa, under controlled environmental conditions (temperature: 24-25°C, humidity: 60-65%, and a 12-hour light-dark cycle) for two weeks prior to the procedure. All rats were subjected to midline laparotomy incision to harvest the left kidney under general anesthesia (intraperitoneally injection of 100 mg/kg ketamine and 10 mg/kg xylazine) [16]. The rats were divided randomly into four groups (6 rats in each group) as follows:

1. **Sham group:** Rats were subjected to previously mentioned procedures without induction of renal ischemia.
2. **Control group:** Rats were subjected to bilateral renal arterial ligation and ischemia for 30 minutes, followed by 2 hours of reperfusion [17].
3. **Vehicle group:** Rats were injected intraperitoneally with dimethyl sulfoxide (DMSO) about 24 hrs before renal ischemia as a vehicle for NHWD-870, followed by 2 hours of reperfusion (Med Chem express/USA).
4. **NHWD-870 group:** Rats were orally administered 3 mg/kg of NHWD-870 24 hours before undergoing ischemia, after which the rats were sedated and subjected to 30 minutes of bilateral renal ischemia followed by a 2-hour reperfusion period [18].

### Experimental procedure

Rats were put on their back, and their limbs and tails were fixed with a medical plaster after they were anesthetized with ketamine and xylazine hydrochloride. A midline laparotomy incision was made to expose the abdomen and the right and left renal pedicles. Then, bilateral ligation of the renal artery for 30 min. was performed, followed by reperfusion for 2 hrs. To prevent dehydration, 1 cc of an isotonic sodium chloride 0.9% solution warmed to 37 °C was applied to the abdomen. After a two-hour reperfusion period, blood samples were collected via cardiac puncture, and the left kidney was removed for laboratory analysis. The collected samples were used for the assessment of inflammatory, oxidative, and apoptotic markers, as well as for histological analysis [15]. It is important to note that kidney damage can occur if ischemia lasts longer than 20 minutes, and reperfusion after ischemia exceeding 40 minutes can lead to irreversible damage.

### NHWD-87 preparation

The left kidney tissue sample was washed in xylene, embedded in a paraffin block, and fixed in 10% formalin. The tissue was then dehydrated using an alcohol series and sliced horizontally to achieve a thickness of approximately 5 micrometers. The resulting tissue slide sections were stained with hematoxylin and eosin (H&E) dye, which allowed for the visualization of cellular structures and tissue abnormalities. These stained slides were subsequently sent to a histopathologist for evaluation. To assess the degree of renal damage, a histological scoring system was employed. Slides were examined at a magnification of X40 and assigned a score based on the percentage of renal tubular damage observed [19]. The scoring system was as follows:

Score 0: represents a normal condition

Score 1: represents < 25% of tubules being damaged

Score 2: represents 25%-50% of tubules being damaged

Score 3: represents 50% -75% of tubules being damaged

Score 4: represents >75% of tubules being damaged [20].

### Serum assays

Serum assays were performed to assess blood urea nitrogen (BUN) levels and serum creatinine as indicators of renal function. A volume of 2 mL of blood was collected through aspiration to assess these parameters. The levels of BUN and serum creatinine were determined using spectrophotometric assays. All kits used in the study were utilized in accordance with the guidelines provided by the manufacturer (Nanjing Jincheng Co., China).

### Preparation of tissue for measurement of IL-1B, PGF-2, PI3K/AKT, Bcl-2 by ELISA

Following the surgical procedure, the left kidney of the rats was carefully extracted and rinsed with ice-cold normal saline solution to remove any residual blood clots and then divided into two halves. High-intensity ultrasonic liquid processing was used to homogenize one segment in phosphate-buffered saline that was 1:10 (w/v) diluted and contained 1% Triton X-100 and a protease inhibitor cocktail [21]. The homogenate was centrifuged for 20 minutes at 4°C22 at 14000 rpm [22]. The supernatant was collected to analyze IL-1B, Bcl-2, PGF-2 isoprostane, and PI3K/AKT using the ELISA method.

### Statistical analysis

Graph Pad Prism was used for the statistical analysis (version 9). The information was presented as mean ± SEM. For multiple comparisons, analysis of variance (ANOVA) and the LSD post-hoc test were utilized. Mann Whitney U and the Kruskal-Wallis test were used to analyze the histopathological data. p-value was considered significant at p≤0.05.

## RESULTS

### NHWD-870 effect on renal function test

**Figure 1:**
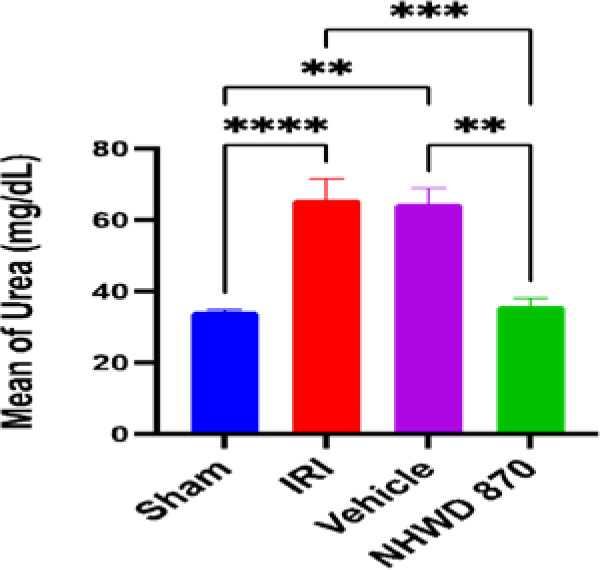
Mean serum level of urea (mg/dl) across the four experimental groups **Significant difference sham vs. vehicle, NHWD-870 vs. vehicle p-value<0.01 *** Significant difference NHWD-870 vs. IRI p-value<0.001 **** Significant difference sham vs. IRI p-value<0.0001

**Figure 2:**
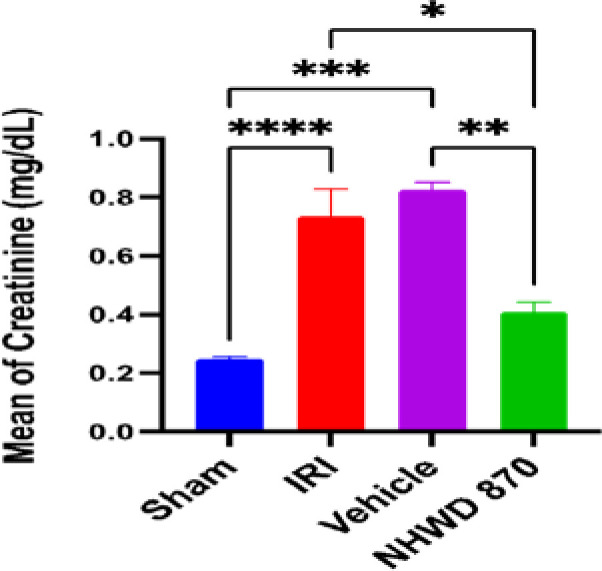
Mean serum level of creatinine (mg/dl) across the four experimental groups *Significant difference NHWD-870 vs. IRI p-value<0.01 **Significant difference NHWD-870 vs. vehicle p-value<0.001 ***Significant difference sham vs. vehicle p-value<0.0001 ****Significant difference sham vs. IRI p-value<0.00001

Serum urea and creatinine levels were significantly increased in the control and vehicle groups compared to the sham group. However, pretreatment with NHWD-870 significantly reduced the levels of these markers compared to the control and vehicle groups (Figure 1 and Figure 2).

### NHWD-870 effect on inflammatory marker IL-1B in renal tissue

**Figure 3:**
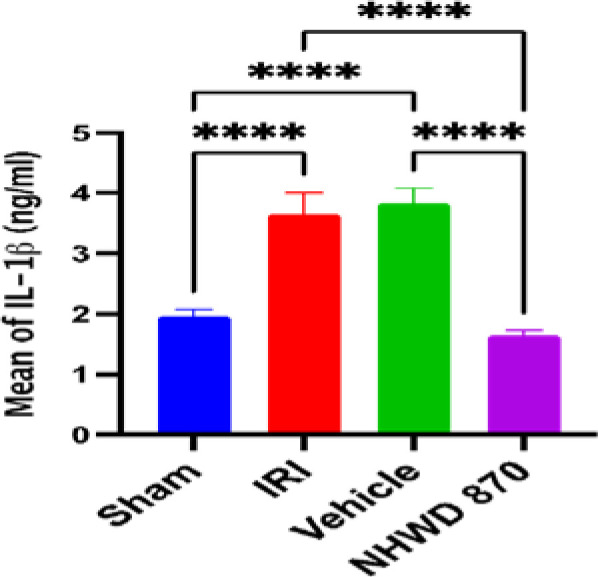
Mean renal tissue level of IL-1B (ng/ml) across the four experimental groups **** Significant difference sham vs. IRI, sham vs. vehicle, NHWD-870 vs. IRI, NHWD-870 vs. vehicle p-value<0.0001

The renal tissue level of IL-1B was significantly higher in the control and vehicle groups than in the sham group. However, the NHWD-870 pretreated group had a significant decrease in the protein expression of inflammatory mediator IL-1B as compared with control and vehicle groups (Figure 3).

### NHWD-870 effect on oxidative stress marker PGF-2 isoprostane in renal tissue

**Figure 4:**
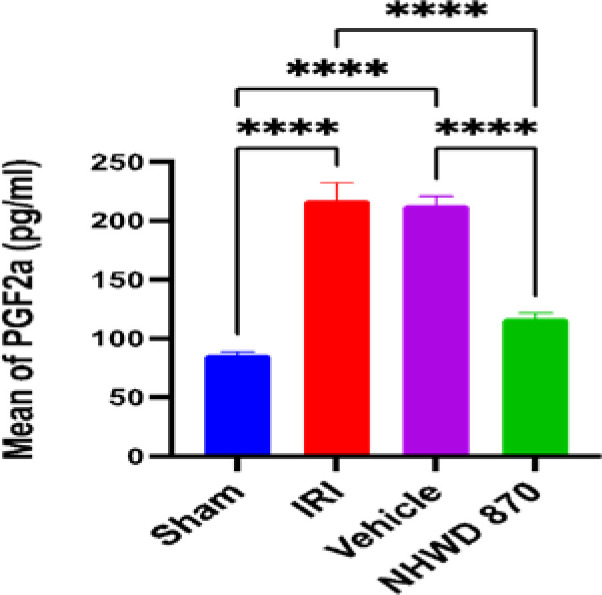
Mean renal tissue of PGF2 (pg/ml) across the four experimental groups **** Significant difference sham vs. IRI, sham vs. vehicle, NHWD-870 vs. IRI, NHWD-870vs vehicle p-value<0.0001

The renal tissue level of PGF-2 was significantly increased in the control and vehicle groups compared to the sham group. However, pretreatment with NHWD-870 significantly decreased the renal tissue level of PGF-2 compared to the control and vehicle groups (Figure 4).

### NHWD-870 effect on apoptotic marker Bcl-2 in renal tissue

**Figure 5:**
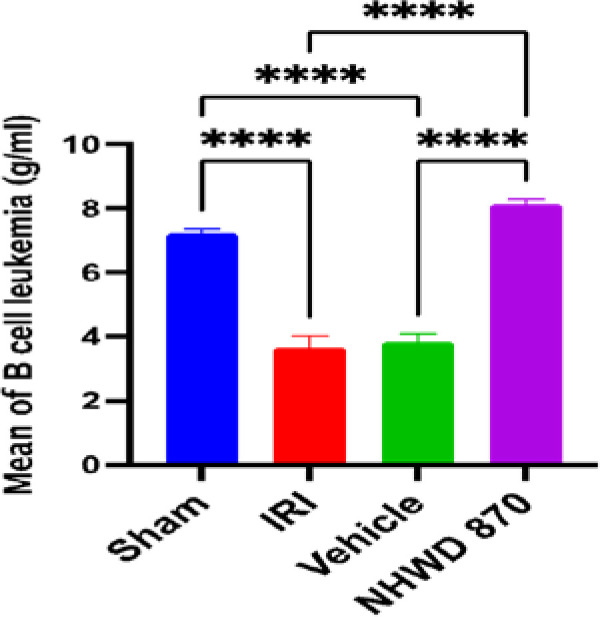
Mean renal tissue of Bcl-2 (g/ml) across the four experimental groups **** Significant difference sham vs. IRI, sham vs. vehicle, NHWD-870 vs. IRI, NHWD-870 vs. vehicle p-value<0.0001

Rats in the control and vehicle groups exhibited a significant decrease in renal tissue level of Bcl-2 compared to the sham group. NHWD-870 pretreatment significantly increased the renal tissue level of Bcl-2 compared to the control and vehicle groups (Figure 5).

### NHWD-870 effect on PI3K/AKT expression

**Figure 6:**
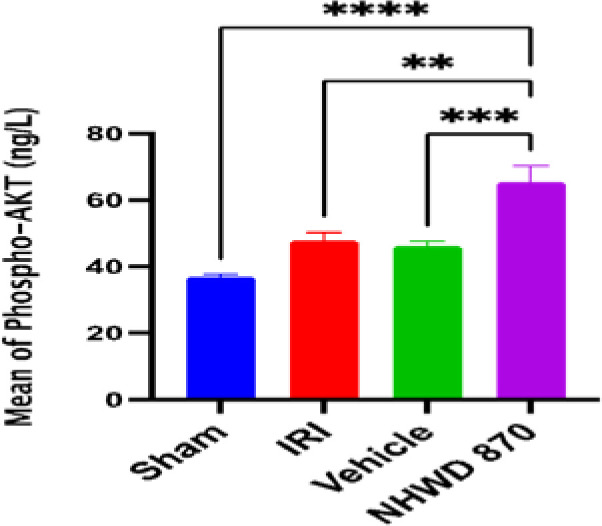
Mean renal tissue of PI3K-AKT (ng/L) across the four experimental groups **Significant difference NHWD-870 vs. IRI p-value<0.01 ***Significant difference NHWD-870 vs. vehicle p-value<0.001 ****Significant difference NHWD-870 vs. sham p-value<0.0001

**Figure 7:**
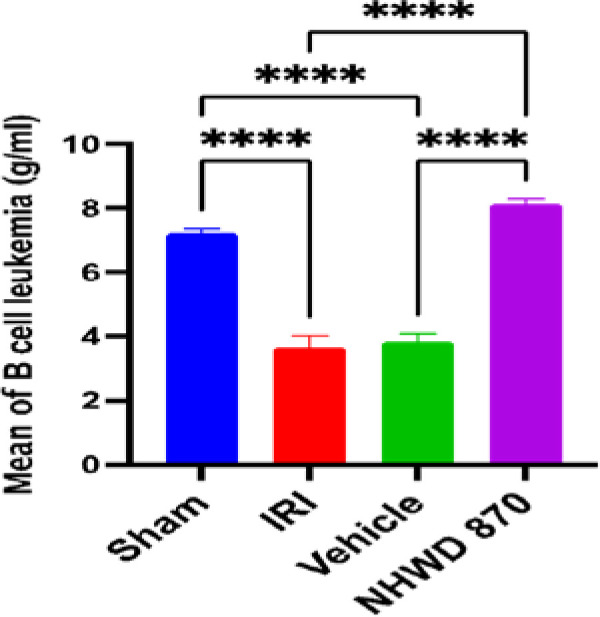
Mean renal tissue of PI3K-AKT (ng/L) across the four experimental groups ****significant difference sham vs. IRI, sham vs. vehicle, NHWD-870 vs. IRI, NHWD-870 vs. vehicle p-value<0.0001

The protein expression of PI3K /AKT was significantly higher in the sham groups compared to the control and vehicle groups. On the other hand, treatment with NHWD-870 significantly increased the protein expression of PI3K /AKT (Figures 6 and 7).

### NHWD-870 effect on kidney injury

**Figure 8:**
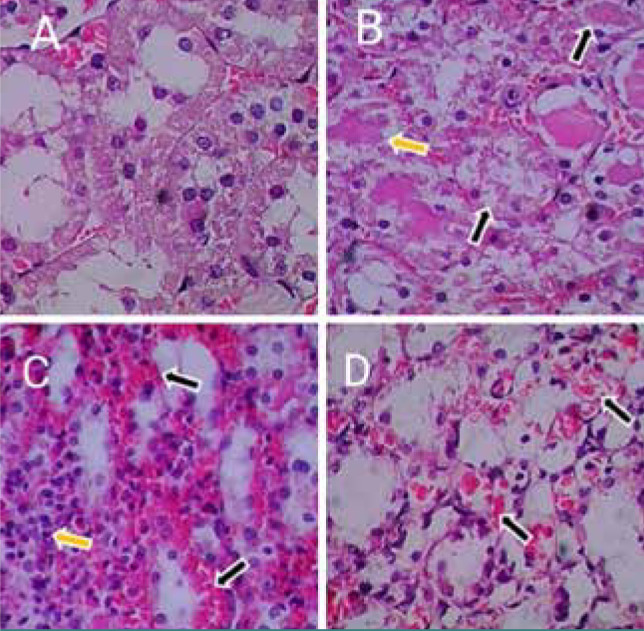
Histopathological examination of renal section. A: photomicrograph of the renal section for the sham group, showing normal histology of renal tubules (score= 0). H&E staining, magnification 400x B: photomicrograph of the renal section for the control group, demonstrating severe renal tissue damage (score=4). Cellular swelling, increased cytoplasmic eosinophilia, loss of brush border, and presence of eosinophilic casts and hemorrhage (black arrows) surrounded renal tubules and glomerulus and occupied spaces of the necrotic renal tubule, H&E, (400X), C: photomicrograph of the renal section for the vehicle group, showing extensive damage to renal tubules (score=4). Diffuse cellular swelling, increased cytoplasmic eosinophilia, loss of brush border, and hemorrhage/ necrosis of epithelial cells (black arrows) of renal tubules of cortex area, where the inflammatory exudate (yellow arrows) was observed in the lumen of renal tubules under necrosis, H&E (400X), D: photomicrograph of the renal section for the NHWD treatment group, showing moderate damage (score=2) affecting 40% of renal tubules. Features included cellular swelling, increased cytoplasmic eosinophilia, and hemorrhage. H&E (400x).

Histopathological examination showed mild tubular injury in the kidney of the sham group. Compared with control and vehicle groups, an increased number of damaged tubules and tubular dilatation were noticed compared to the sham group. NHWD-870 pretreated group showed little histological change compared to the control and vehicle groups (Figure 8 A-D).

## DISCUSSION

Renal ischemia-reperfusion (IRI) is the primary cause of acute kidney damage (AKI) associated with significant morbidity and mortality. Various studies are directed toward investigating the potential nephroprotective roles of different drugs, chemicals, and natural and synthetic products to improve the clinical outcome of AKI and reduce the degree of renal damage. NHWD-870, a specific Brd4 inhibitor, is known to have favorable pleotropic features, inhibits apoptosis and decreases pro-inflammatory markers production, and has antioxidant properties, making it a promising therapeutic target in renal ischemia-reperfusion injury. To our knowledge, no previous study investigated the nephroprotective role of NHWD-870 in renal IRI.

### Effect of NHWD-870 on renal function parameters (urea and creatinine)

NHWD-870 significantly decreased the levels of urea and creatinine compared to the control group and vehicle groups representing an improvement in renal function following renal IRI induction. However, no prior studies have specifically examined the effect of NHWD-870 on serum urea and creatinine levels in the context of renal IRI. These findings support the potential of NHWD-870 as a therapeutic intervention to protect against renal injury in ischemic conditions.

### Effect of NHWD-870 on inflammatory mediators

NHWD-870 pretreatment also resulted in a significant decrease in the level of the oxidative stress marker F2-isoprostane in renal ischemic tissues compared to the control and vehicle groups. This finding suggests that NHWD-870 has antioxidant properties in kidney tissues subjected to ischemia and reperfusion. However, there is a lack of previous research investigating the effects of NHWD-870 on pro-inflammatory mediators such as IL-1B.

### Effect of NHWD-870 on oxidative stress marker (F2-isoprostane)

NHWD-870 pretreatment also resulted in a significant decrease in the level of the oxidative stress marker F2-isoprostane in renal ischemic tissues compared to the control and vehicle groups. This finding suggests that NHWD-870 has antioxidant properties in kidney tissues subjected to ischemia and reperfusion. However, there is currently no available literature describing the effect of NHWD-870 on the oxidative stress marker PG F2-isoprostane. These results imply an antioxidant and nephroprotective effect of NHWD-870.

### Effect of NHWD-870 on apoptotic marker Bcl-2

In contrast to the control and vehicle groups, pretreatment with NHWD-870 prior to the induction of ischemia significantly increased the level of Bcl-2 in the pretreatment group. This indicates that NHWD-870 has an anti-apoptotic effect in renal IRI. However, there is no previous study describing the effect of NHWD-870 on the Bcl-2 anti-apoptotic marker.

### Effect of NHWD-870 on PI3K/AKT signaling pathway

Furthermore, NHWD-870 treatment led to a significant increase in the levels of the PI3K/AKT signaling pathway in renal tissues. This finding suggests that NHWD-870 exerts nephroprotection against renal IRI by activating the PI3K survival pathway. Another study [17] demonstrated the activation of PI3K/AKT1 and ERK1 in tumor-associated macrophages (TAM) induced by co-culturing them with A2780 cells in vitro. Moreover, pretreating A2780 cells with NHWD-870 reduced CSF-1 production in tumor cells. These findings suggest that NHWD-870 may have direct macrophage-specific anti-proliferative actions.

### Effect of NHWD-870 on renal parenchyma

Pretreatment of rats with the selective Brd4 inhibitor NHWD-870 before the onset of ischemia considerably reduced kidney damage severity and restored nearly normal renal tissue architecture compared to renal injury severity in both the control and vehicle groups. These findings are consistent with earlier studies that have reported the renoprotective effects of NHWD-870 in mitigating renal injury [22]. BET inhibitors have been demonstrated to block a number of transcription factors, including c-Myc and p53, therefore slowing the development of malignancies. Many studies showed that NHWD-870 treatment significantly decreased the amount of c-Myc and p53 expression, suggesting that this may be one method NHWD-870 inhibits renal fibrosis and necrosis [23-25].

## CONCLUSION

NHWD-870 demonstrates nephroprotective effects in renal ischemia-reperfusion injury by reducing the inflammatory response and oxidative stress (as indicated by decreased IL-1B and F2-isoprostane). Additionally, NHWD-870 inhibits apoptosis through the upregulation of the anti-apoptotic protein Bcl-2, achieved via the activation of the PI3K/AKT signaling pathway.

## Personal thanks

We would like to extend our sincere appreciation to the Department of Pharmacology and the Central Research Unit of the Faculty of Medicine, Kufa University, as well as the College of Science, Kufa University, for their invaluable assistance and support throughout this study.
